# The association of the MIND diet and its components with cognitive function in postmenopausal breast cancer survivors

**DOI:** 10.1007/s00520-025-09789-9

**Published:** 2025-07-31

**Authors:** Timothy R. Winschel, Kellie Weinhold, Patrick M. Schnell, Stephanie Gorka, Maryam Lustberg, Darrin Aase, Zihan Melink, Rachel Kopec, Chase Picino, Ziqi Li, Bridget Oppong, Nicole Williams, Tonya S. Orchard

**Affiliations:** 1 Human Nutrition Program, Department of Human Sciences, The Ohio State University, Columbus, OH 43210 USA; 2 Division of Biostatistics, College of Public Health, The Ohio State University, Columbus, OH 43210 USA; 3 Department of Psychiatry and Behavioral Health, The Ohio State University Wexner Medical Center, Columbus, OH 43210 USA; 4 Yale School of Medicine, Center for Breast Cancer, New Haven, CT 06511 USA; 5 Foods for Health Discovery Theme, The Ohio State University, Columbus, OH 43210 USA; 6 College of Pharmacy, The Ohio State University, Columbus, OH 43210 USA; 7 Department of Food Science and Technology, The Ohio State University, Columbus, OH 43210 USA; 8 Department of Surgery, The Ohio State University Wexner Medical Center, Columbus, OH USA; 9 Department of Medical Oncology, The Ohio State University Wexner Medical Center, Columbus, OH USA

**Keywords:** Breast cancer, MIND diet, Cancer related cognitive impairment, Chemotherapy

## Abstract

**Purpose:**

Cancer related cognitive impairment is frequently reported by cancer survivors. The purpose of this study was to determine the association of the MIND diet pattern and its components with cognitive function in breast cancer survivors following chemotherapy.

**Methods:**

This was a cross-sectional study of 30 post-menopausal breast cancer survivors within one year of completing chemotherapy; 21 had complete data for analysis. Cognitive outcomes were assessed using a battery of objective tests. Self-reported dietary data were collected through electronic food frequency questionnaire; nutritional biomarkers were measured in blood. Linear regression models assessed the associations between the MIND diet score and its components with cognitive outcomes.

**Results:**

Adherence to the MIND diet pattern was positively associated with global cognitive score (β = 1.163, *p* = 0.013), episodic memory (β = 3.726, *p* = 0.019), attention/working memory (β = 4.046, *p* = 0.024) and phonemic fluency (β = 42.305, *p* = 0.002) adjusting for education and total energy intake. Foods emphasized in the MIND diet such as non-fried fish, vegetables, and beans/legumes were all positively associated with cognitive performance on individual tests (*p* < 0.05 for all). Blood biomarkers of vegetable (β-carotene) and non-fried fish (docosahexaenoic acid (DHA)) correlated with self-reported intake. Blood DHA was correlated with better cognitive performance (*p* < 0.05) Additionally, moderate intake of red meat and lower intake of fast food were positively associated with cognitive performance (*p* < 0.05 for both).

**Conclusions:**

Among post-menopausal breast cancer survivors that are within one year of completing chemotherapy, adherence to the MIND diet pattern and its components was associated with better cognitive function.

**Supplementary Information:**

The online version contains supplementary material available at 10.1007/s00520-025-09789-9.

## Introduction

Side effects from cancer or cancer treatments that impact cognitive function are referred to as cancer related cognitive impairment (CRCI). Prevalence of CRCI is estimated to be as high as 75% during active treatment for cancer [1], and is reported by approximately one-third of breast cancer survivors that have completed chemotherapy [2]. The International Cognition and Cancer Task Force (ICCTF) recommends measuring cognitive function in cancer survivors using a battery of neuropsychologic tests that target the main cognitive domains (i.e., executive function, attention, verbal fluency, learning, and memory) impacted by cancer and cancer treatment [3, 4]. Declines in cognitive function contribute to negative impacts on quality of life for cancer survivors [5–7].

The underlying mechanisms behind CRCI are an area of active research, with oxidative stress and neuroinflammation being two probable contributing factors [2, 3]. Diet and nutrition interventions have the potential to act as preventative and management strategies for both of these mechanisms [6, 8, 9]. The Mediterranean-DASH Intervention for Neurodegenerative Delay (MIND) diet pattern was developed based on food groups associated with neuroprotection [10]. The diet pattern is comprised of ten “brain healthy” foods and five “brain unhealthy” foods, with a focus on increasing essential micronutrients such as vitamins E, B9, B12, D; unsaturated essential fatty acids like docosahexaenoic acid (DHA); and a variety of antioxidants such as polyphenols and carotenoids [10]. While the MIND dietary pattern has been associated with reductions in cognitive impairment and neurodegenerative diseases [11–14], the relationship between the MIND diet pattern and CRCI in cancer survivors is still unclear.

Therefore, the aim of this study was to investigate the association of the MIND dietary pattern and its components with cognitive function in postmenopausal breast cancer survivors that were within 1-year post-completion of chemotherapy. We hypothesized that greater adherence to the MIND diet pattern and its recommended components would be associated with better cognitive functioning.

## Methods

### Design and sample

This study was a cross-sectional observational study conducted at The Ohio State University (OSU). Complete data was collected on 21 participants. Eligible subjects were post-menopausal females aged 45–75, 3–12 months post completion of chemotherapy treatment, diagnosed stage I-III breast cancer, and were English speaking. Exclusion criteria included contraindications to magnetic resonance imaging (MRI), pregnant or breastfeeding, concurrent other malignancy or metastatic malignancy, and diagnosed with alcohol or substance disorders, stroke, dementia, or psychosis.

### Recruitment and enrollment

Eligible survivors were recruited from electronic medical record review, online newsletters, and online or printed study flyers. Recruitment occurred from January 2022 to June 2023. All enrolled subjects provided electronic written informed consent and HIPPA authorization. Participants all lived within the state of Ohio at the time of the study and were patients at the OSU Wexner Medical Center. The OSU Cancer Institutional Review Board approved the study protocol. The study is listed at ClinicalTrials.gov with identifier NCT05122000.

### Data collection

This study included remote and in-person visits. Research Electronic Data Capture (REDCap) was used to collect responses to questionnaires related to demographic and clinical characteristics of participants.

#### Dietary data

Dietary data were collected using the electronic food frequency questionnaire platform, VioScreen® (version: 2.99.2.762). VioScreen® data reflect intake over the previous 3 months [15]. The MIND diet score was calculated from 14 dietary components: 9 foods to promote neuroprotection and 5 foods to limit. Wine was removed from the calculation of the MIND score as limiting or avoiding alcohol is recommended for cancer survivors [4, 16, 17]. The maximum possible MIND diet score of 14 indicates optimal intake of all 14 dietary components; scoring cutoffs can be found in Supplemental Table 1.

#### Blood nutritional biomarkers

To address biases related with self-reported dietary intake, nutritional biomarkers [carotenoids from plasma and polyunsaturated fatty acids (PUFAs) from dried blood spots (DBS)] were also measured. We hypothesized that DBS PUFA levels would be reflective of non-fried fish as this is a well-established correlation [18]. We hypothesized that the plasma carotenoids would be less likely to reflect strong correlations with the green leafy and other vegetables categories of the MIND pattern due to the wide distribution of carotenoids in plant foods, some of which are not included in the MIND pattern, as well as limited bioavailability of carotenoids consumed in the diet [19]. During an in-person clinic visit, venous whole blood was collected by a trained phlebotomist and processed. To separate blood fractions samples were spun at 1200 g for 10 min at 4 °C. DBS collection materials were mailed to participants, including one card that was treated with butylated hydroxytoluene to protect polyunsaturated fatty acids from degradation. Samples were returned through postal mail to the research lab. Both sample types were stored at −80 °C.

Plasma carotenoids were spiked with a retinyl acetate solution (5 μL) used as an internal standard (IS). Plasma samples (300 μL) were then extracted using a biphasic method published previously [20], except the extraction solvent consisted exclusively of methyl-tert butyl ether. Extracts were dried under argon and stored at −80 °C not more than 1 week prior to reconstitution and analysis via liquid chromatography-photodiode array detection (Agilent 1200 series, Santa Clara, CA). Separations were achieved using the previously published gradient and column [21]. Sample injection volume was 10 μL, and the column was held at 45 °C. External standard curves of α-tocopherol, lutein, zeaxanthin, β-cryptoxanthin, retinol, α-carotene, β-carotene, and lycopene were used to quantitate the analytes in the blood plasma samples, corrected against the IS.

Analysis of DBS fatty acids was achieved using gas-chromatography. In brief, fatty acids were extracted and methylated using boron trifluoride, following previous protocols [22]. Fatty acid methyl esters were analyzed using a gas chromatograph (Shimadzu Scientific Instruments, Columbia, MD, USA) with 30-m Omegawax TM 320 fused silica capillary column (Supelco, Bellefonte, PA, USA). Retention times were evaluated against standards (Nu‐Check Prep Inc., Elysian, MN, USA). Fatty acids are reported as percent of total fatty acids. PUFAs biomarkers of specific interest included the marine derived omega-3 fatty acids, eicosapentaenoic acid (EPA) and docosahexaenoic acid (DHA).

Self-reported dietary intake was used as primary predictor of cognitive function. Nutritional biomarkers from the previously described methods were used to support dietary intake and were not included as individual predictors of cognitive function as not all the MIND diet components can be represented by nutritional biomarkers in the blood.

#### Cognitive function

Cognitive function was assessed during a 30-min virtual Zoom ® session with trained study staff in which a battery of neuropsychologic tests, aligning with ICCTF recommendations, were administered [4]. Staff were trained by study neuropsychologist on administration of virtual testing [23]. The cognitive battery included the Hopkins Verbal Learning Test – Revised (HVLT-R), the digit span component of the Wechsler Adult Intelligence Scale-IV (WAIS-IV), the Oral Trail Making Test (OTMT), and the Controlled Oral Word Association Test (COWAT).

The HVLT-R is a test of episodic memory. Trials 1–3 are an assessment of immediate recall, while trial four is a measure of delayed recall [24]. The digit span forward and digit span backward tasks of the WAIS-IV assess cognitive domains of attention and working memory [25]. Trial A of the OTMT measures processing speed and attention while Trial B assesses executive function [26]. The COWAT is a measure of verbal fluency, with a trial of letters (FAS) measuring phonemic fluency and a trial focused on a specific category (animals) measuring semantic fluency [27].

In accordance with a recent randomized control trial of the MIND diet [28], raw scores from each test were converted to z-scores using the sample mean and standard deviation. Values from the OTMT were reverse coded to align with all other tests where a higher score represented better performance. A global cognitive score was calculated by averaging z-scores from the 8 individual tests (HVLT-R trials 1–3, HVLT-R trial 4, digit span forward, digit span backward, OTMT trial A, OTMT trial B, COWAT FAS, COWAT Animals). Higher global cognitive scores indicate better cognitive function.

### Statistical analysis

Demographic and clinical data are presented as mean (standard deviation) and frequency (percentage) for continuous and categorical variables respectively. A two-sample t-test assuming equal variance was conducted to assess whether there were significant differences in mean cognitive test scores between the study sample and normative data. Spearman correlation analyses were performed to examine the relationship between nutritional biomarkers and self-reported intake of corresponding foods. For biomarkers found to be significant in the initial analyses, additional Spearman correlations were conducted to evaluate their associations with performance on cognitive tests.

The primary analysis assessed the association between adherence to the MIND diet pattern score with cognitive function measured by the global cognitive score adjusted for education and total energy intake. Secondary analyses included evaluating the association between adherence to the MIND diet and its components with cognitive function adjusted for total energy intake and education level. Exploratory analyses included a median split of the MIND diet scores into a low and high category to allow for comparison of demographic and clinical characteristics via two-sample t-test for continuous variables and Fisher’s exact test for categorical variables.

Linear regression was used to assess both primary and secondary analyses. Due to small sample size, models were limited in the number of covariates included, therefore we chose to include education level and total energy as the primary covariates of interest in the relationship between diet and cognitive function because of potential confounding of these variables. Education level was collapsed into two groups, obtained a bachelor’s degree or advanced degree (*n* = 10) or did not (*n* = 11). Assumptions of linear regression models were assessed through residual analysis. The Benjamini-Yekutieli procedure [29] was chosen to adjust for the false discovery rate across the secondary outcomes due to validity in the presence of dependencies between MIND diet components and the overall MIND diet score, as well as between the global cognitive score and individual test scores [29]. Dietary predictors were transformed using log(x + a) transformation with a = (90th percentile/10) to handle zeroes adaptively in response to the original scales of the variables. All p-values and confidence intervals were two-sided, with a significance threshold of *p* < 0.05. Analyses were conducted using Stata/SE 18.0 (StataCorp LLC, College Station, TX, USA).

## Results

### Recruitment and demographic/clinical characteristics

The flow diagram (Supplemental Fig. 1) describes recruitment and enrollment of the study. There were 118 potential participants screened and 30 enrolled. Of the 30 participants, 21 had complete data sets and were included in analyses.

Demographic and clinical characteristics are presented in Table 1. Participants enrolled in the study had a mean age of 58 years (SD = 7.44) with a range of 45–69 years. The sample identified primarily as white (90.48%), with 47.62% reporting a bachelor’s degree or advanced degree, and worked full time (47.62%). There was a low level of physical activity among participants, with the majority (76.19%) self-reporting either low activity or sedentary levels. Clinically, stage II breast cancer diagnosis was the most common (*n* = 9, 42.86%) with stages I and III both had 6 participants (28.57%). The majority of participants reported positive HER-2 status (66.67%). Per inclusion criteria, all participants received chemotherapy as part of their breast cancer treatment regimen. Supplemental Table 2 provides results from our exploratory analysis of differences between high and low MIND scores on demographic and clinical characteristics. We observed that participants with lower MIND scores more frequently reported working full time or part time as well as positive HER2 status compared to those in the high MIND score group (*p* < 0.05).

**Table 1:** Demographic and Clinical Characteristics^a^

Variable	Total
	Mean (SD)
Age (years)	58.00 (7.44)
Race	N (%)
Black or African American	2 (9.52)
White	19 (90.48)
Education	N (%)
Less than a bachelor’s degree	11 (52.38%)
Bachelor’s degree or advanced degree	10 (47.62%)
Employment	N (%)
Work 40 + hours a week	10 (47.62)
Work fewer than 40 h a week	1 (4.76)
Homemaker	2 (9.52)
Retired	3 (14.29)
Unemployed	5 (23.81)
Physical Activity Level	N (%)
Sedentary	6 (28.57)
Low Active	10 (47.62)
Active	5 (23.81)
Very Active	0 (0.00)
Extremely Active	0 (0.00)
Breast Cancer Stage	N (%)
I	6 (28.57)
II	9 (42.86)
III	6 (28.57)
HER-2 Status	N (%)
Positive	6 (28.57%)
Negative	14 (66.67%)
Unknown	1 (4.76%)
Treatment ^b^	N (%)
Surgery—lumpectomy/partial mastectomy	5 (23.81%)
Surgery—total (simple) mastectomy	5 (23.81%)
Surgery—modified radical mastectomy	11 (52.38%)
Radiation therapy	14 (66.67%)
Chemotherapy	21 (100.00%)
Anti-Hormone or anti-estrogen therapy (e.g. Tamoxifen, Megace, Arimidex, Femara or other aromatase inhibitor)	9 (42.86%)
Targeted therapy (e.g. HER2 inhibitors [Herceptin], CDK4/6 inhibitors [Ibrance], etc.)	6 (28.57%)
Immunotherapy (e.g. Keytruda, Tecentriq)	7 (33.33%)

^a^*HER2* human epidermal growth factor receptor 2, *CDK* cyclin-dependent kinase

^b^Treatment categories are not mutually exclusive, therefore total percentages sum to greater than 100%

### Dietary characteristics

Descriptive results of the dietary predictors are presented in Table 2. The mean MIND diet score among the 21 participants was 6.60 (SD = 1.59, range 4–11) out of 14. Among “brain healthy foods”, mean intake of other vegetables, [21.06 (13.29) servings per week], and non-fried fish, [1.09 (1.69) servings per week], met the recommended servings of the MIND diet pattern [28]. The mean intake of red and processed meat, 2.15 (1.13) servings per week, and pastries and sweets, 3.76 (3.60) servings per week, met the recommended servings of the MIND diet pattern [28].

### Neuropsychologic tests and CRCI characteristic

Higher scores of global cognition indicate better overall cognitive function. Among the individual tests, higher scores indicate better performance, except for OTMT-A and OTMT-B, where lower scores indicate better performance. Table 2 presents the sample mean, standard deviation, and range for the eight individual cognitive tests and global cognitive score, as well as normative means for healthy female adults. The mean global cognitive score was 0.15 (0.54). Sample means of the HVLT trials 1–3 (27.38 words) and HVLT trial 4 (10.29 words) were similar to the normative means for each respective test (27.5 words and 9.8 words). The participants performed better on tasks of digit span forward 10.62 (1.88) and digit span backwards 9.33 (1.93) compared to normative means. Participants performed worse on OTMT-A compared to healthy normative mean (sample mean: 7.53(1.22) seconds, normative mean: 6.88(1.89) seconds), while they performed better on OTMT-B (sample mean: 28.72(11.70) seconds, normative mean: 33.91(17.58) seconds). Performances on FAS trial and Animals trial of the COWAT were similar between the sample (FAS: 45.10, Animals: 22.52) and normative data (FAS: 44.7, Animals: 21.9). Means were similar among the test comparisons, except that the sample had significantly higher scores on the test of DSB compared to normative means indicating better performance.

**Table 2:** MIND diet components, nutritional biomarkers and cognitive function summary variables^a^

MIND Diet Variables				
Component	Unit	Mean (SD)	(Min, Max)	Serving Recommendation ^b^
Green Leafy Vegetables	servings/week	2.85 (2.02)	(0.00, 6.37)	≥ 7
Other Vegetables	servings/week	21.06 (13.29)	(4.55, 51.78)	≥ 7
Berries	servings/week	2.20 (2.46)	(0.00, 8.85)	≥ 5
Nuts	servings/week	3.26 (3.84)	(0.00, 16.07)	≥ 5
Olive Oil	Tbsp/day	0.21 (0.30)	(0.00, 1.22)	≥ 2
Whole Grains	servings/day	1.44 (1.41)	(0.00, 5.46)	≥ 3
Non-fried Fish	servings/week	1.09 (1.69)	(0.00, 7.06)	> 1
Beans and Legumes	servings/week	1.45 (1.32)	(0.00, 4.68)	≥ 3
Poultry (not fried, skinless)	meal/week	1.30 (1.68)	(0.00, 7.86)	≥ 2
Butter and stick margarine	Tsp (pat)/day	1.24 (1.26)	(0.00, 4.35)	≤ 1
Regular Cheese	servings/week	6.62 (4.41)	(1.31, 17.09)	≤ 2
Red Meat and Processed Meats	servings/week	2.15 (1.13)	(0.25, 4.22)	< 4
Fast and Fried Foods	meals/week	2.68 (2.62)	(0.00, 9.47)	< 1
Pastries and Sweets	servings/week	3.76 (3.60)	(0.00, 12.24)	< 5
MIND Diet Score	score	6.60 (1.59)	(4.00, 11.00)	14
Nutritional Biomarkers				
Biomarker^c^	Unit	Mean (SD)	(Min, Max)	N^d^
α-tocopherol	µmol/L	14.60 (6.16)	(6.30, 33.37)	20
Lycopene	µmol/L	0.17 (0.14)	(0.02, 0.49)	18
β-cryptoxanthin	µmol/L	0.11 (0.09)	(0.05, 0.31)	14
Lutein	µmol/L	0.18 (0.14)	(0.05, 0.49)	20
Zeaxanthin	µmol/L	0.08 (0.02)	(0.06, 0.13)	9
β-carotene	µmol/L	0.19 (0.22)	(0.02, 0.92)	18
Retinol	µmol/L	2.73 (0.86)	(1.70, 5.16)	20
EPA (20:5 n-3)	% total fatty acids	0.004 (0.002)	(0.002, 0.011)	21
DHA (22:6 n-3)	% total fatty acids	0.016 (0.007)	(0.007, 0.033)	21
Cognitive Function				
Test	Cognitive Domain	Mean (SD)	(Min, Max)	Normative Means^e^
HVLT Sum Trials 1–3	Episodic memory – Immediate Recall	27.38 (3.37)	(21.00, 33.00)	27.5 (4.3)
HVLT Trial 4	Episodic memory – Delayed Recall	10.29 (1.55)	(6.00, 12.00)	9.8 (1.8)
WAIS-IV DSF	Attention/Working Memory	10.62 (1.88)	(5.00, 13.00)	10.0 (2.8)
WAIS-IV DSB	Attention/Working Memory	9.33 (1.93)	(7.00, 14.00)	8.0 (2.5)^f^
OTMT-A	Processing speed/Attention	7.53 (1.22)	(5.17, 10.18)	6.88 (1.89)
OTMT-B	Executive Function	28.72 (11.70)	(12.07, 58.52)	33.91 (17.58)
COWAT (FAS)	Phonemic Fluency	45.10 (14.48)	(21.00, 73.00)	44.7 (11.2)
COWAT (Animals)	Semantic Fluency	22.52 (5.95)	(11.00, 35.00)	21.9 (5.4)
Global Cognitive Score	Overall Cognitive Function	0.15 (0.54)	(−0.96, 1.25)	NA

^a^*EPA* eicosapentaenoic acid, *DHA* docosahexaenoic acid, *HVLT* Hopkins verbal learning test, *WAIS-IV DSF* Wechsler Adult Intelligence Scale – IV digit span forward, *WAIS-IV DSB* digit span backward, *OTMT-A* oral trail making test trial A, *OTMT-B *oral trail making test trial B, *FAS* controlled oral word association test FAS trial

^b^Serving recommendations are an adaptation of previously reported MIND diet cutoffs [28]

^c^Carotenoids were measured from plasma samples. Omega-3 fatty acids were measured from dried blood spots

^d^Sample numbers lower than 21 indicate that levels were below the level of detection for carotenoids

^e^Normative means represent scores on tests from a healthy female population of similar age to our sample. Source of normative means: HVLT [24], Digit Span [30], OTMT [31], COWAT [32]

^f^Significant difference (*p* < 0.05) **between sample mean and normative mean measured by** two sample t-test assuming equal variance

### Correlation of self-reported dietary recall with nutritional biomarkers

To address potential bias related to self-reporting, we examined Spearman correlation coefficients between the self-reported MIND diet components and their relevant nutritional biomarkers. For instance, associations between blood carotenoids and MIND components that are sources of carotenoids including green leafy vegetables and other vegetables. Additionally, correlations between plasma α-tocopherol with MIND components of nuts and wholegrains were also evaluated. The results from these analyses are presented in Supplemental Table 3. Weekly intake of Other Vegetables was significantly associated with plasma β-carotene levels (rho = 0.480, *p* = 0.045). However, these levels of β-carotene were not associated with cognitive function as seen in Supplemental Table 4. No significant associations were detected between leafy green vegetable intake, nuts, and whole grains with blood carotenoids. The lack of association of these biomarkers with self-reported intake may be due, in part, to the wide distribution of carotenoids in a variety of fruits and vegetables, some not captured in the MIND components. Additionally, multiple samples had undetectable levels of several carotenoids measured: this reduced sample sizes, further limiting ability to detect associations.

Correlations of PUFA biomarkers from DBS with self-reported non-fried fish servings were used to confirm self-report, as blood levels of long-chain omega-3 fatty acids, particularly DHA, are well established markers of fish intake [28]. Weekly servings of non-fried fish were positively associated with DHA (rho = 0.536, *p* = 0.013) and positive, but non-significant trends were found for EPA (rho = 0.420, *p* = 0.059). Blood levels of EPA were not associated with cognitive function; however, levels of DHA were statistically significant with several domains of cognitive function including global cognitive function (rho = 0.234, *p* < 0.001), phonemic fluency (rho = 0.456, *p* = 0.039), and semantic fluency (rho = 0.570, *p* = 0.008) which are the same cognitive domains that were significantly associated with weekly servings of non-fried fish intake. In addition, DBS DHA levels were significantly associated with episodic memory (immediate and delayed recall) as well as executive function (*p* < 0.05). These correlations of nutritional biomarkers with MIND categories of other vegetables and non-fried fish servings lend support to the accuracy of these self-reported food groups.

### Linear association of log transformed MIND diet score with cognitive function

**Fig. 1:**
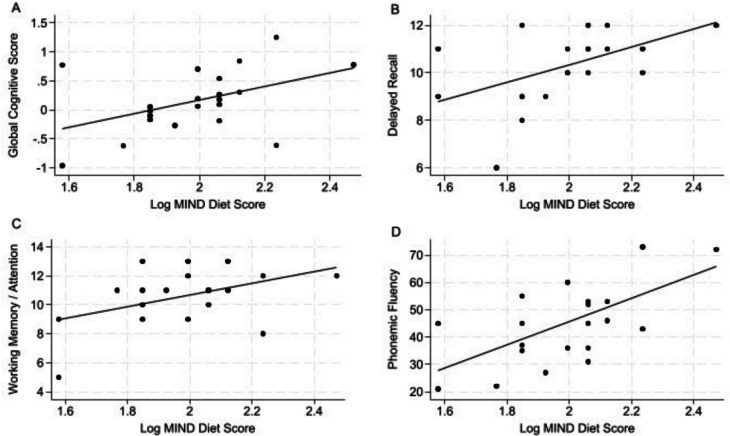
Linear Associations of log transformed MIND diet score with Cognitive Function Adjusted for Education and Total Energy Intake. **A**: Association of MIND diet score with global cognition (β = 1.163, *p* = 0.013). **B**: Association of MIND diet score with episodic memory-delayed recall measured by trial 4 of the HVLT (β = 3.726, *p* = 0.019). **C**: Association of log transformed MIND diet score with working memory/attention as measured by the digit span forward task (β = 4.046, *p* = 0.024). **D**: Association of log transformed MIND diet score with phonemic fluency as measure by the FAS trial of the COWAT (β = 42.305, *p* = 0.002). Figure 1**A** and **D** remain significant following adjustment for multiple comparisons

Figure 1 illustrates the association between log transformed MIND diet pattern score and global cognitive function. The primary analysis is presented in Fig. 1A*;* a positive relationship was found between higher log MIND diet pattern scores and better global cognitive function, adjusting for education level and total energy intake (β = 1.163, *p* = 0.013). Secondary analyses are presented in Fig. 1B-D. In Fig. 1B, greater adherence to the log MIND diet was positively associated with better episodic memory – delayed recall (β = 3.726, *p* = 0.019). Figure 1C shows a positive relationship between higher log MIND diet scores and better working memory (β = 4.046, *p* = 0.024). We observed a positive association between log MIND diet score with phonemic fluency (β = 42.305, *p* = 0.002) in Fig. 1D. Additional results from both unadjusted and adjusted linear regression analyses are available in Supplemental Table 5. Following adjustment for multiple comparisons the association between the MIND diet pattern score with global cognitive function and phonemic fluency remains significant.

### Linear association of log transformed MIND diet components with cognitive function

**Fig. 2:**
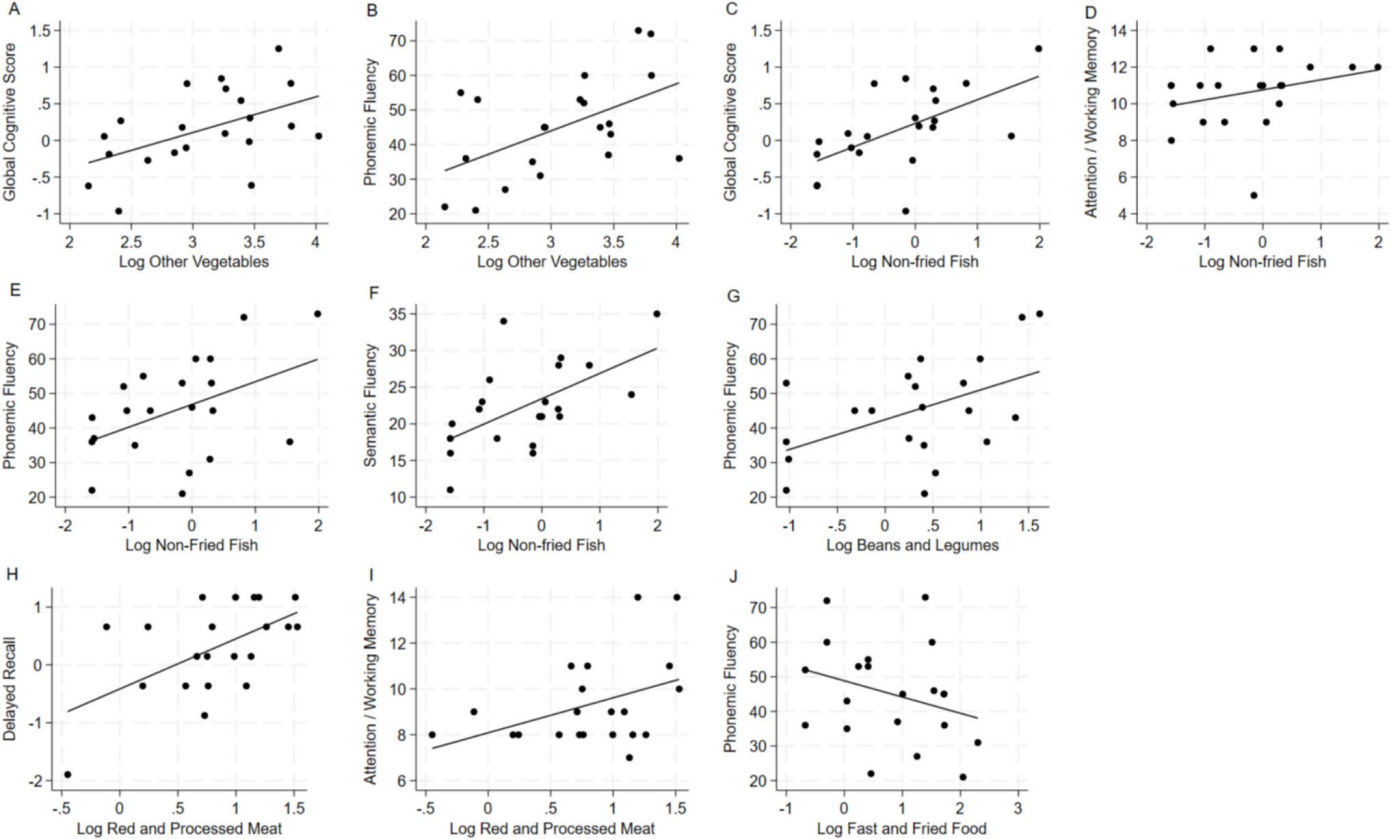
**A**-**G** Linear Association of log transformed “healthy” MIND diet components with Cognitive Function Adjusted for Education and Total Energy Intake. **A**: Association of log transformed servings of other vegetables with global cognitive function (β = 0.410, *p* = 0.039). **B**: Association of log transformed servings of other vegetables with phonemic fluency as measured by the FAS component of the COWAT (β = 14.069, *p* = 0.021). **C**: Association of log transformed servings of non-fried fish with global cognitive function (β = 0.346, *p* = 0.009). **D**: Association of log transformed servings of non-fried fish with attention\working memory as measured by the DSF task (β = 1.150, *p* = 0.025). **E**: Association of log transformed servings of non-fried fish with phonemic fluency as measured by the FAS component of the COWAT (β = 8.778, *p* = 0.039). f: Association of log transformed servings of non-fried fish with semantic fluency as measured by the Animals component of the COWAT (β = 3.731, *p* = 0.027). **G**: Association of log transformed beans and legumes with phonemic fluency as measured by the FAS component of the COWAT (β = 10.168, *p* = 0.016). Figure 2 **H**-**J**: Linear Association of log transformed “un-healthy” MIND diet components with Cognitive Function Adjusted for Education and Total Calorie Intake. **H**: Association of log transformed servings of red and processed meats with episodic memory-delayed recall as measured by the HVLT trial 4 (β = 1.876, *p* = 0.008). **I**: Association of log transformed servings of red and processed meats with attention and working memory as measured by the DSB task of the WAIS-IV (β = 1.876, *p* = 0.035). **J**: Association of log transformed servings of fast and fried food with phonemic fluency as measured by the FAS component of the COWAT (β = −9.408, *p* = 0.024)

Figure 2A-G presents the significant associations between the “brain-healthy” components of the MIND diet and cognitive function. Among “brain healthy” foods, log transformed servings of other vegetables were associated with global cognitive score (β = 0.410, *p* = 0.039) and phonemic fluency (β = 14.069, *p* = 0.021). Log transformed servings of non-fried fish were positively associated with global cognitive score (β = 0.346, *p* = 0.009), attention/working memory (β = 1.150, *p* = 0.025), phonemic fluency (β = 8.778, *p* = 0.039), and semantic fluency (β = 3.731, *p* = 0.027). Log transformed servings of beans and legumes were positively associated with phonemic fluency (β = 10.168, *p* = 0.016).

Figure 2**H**-**J** present the significant associations between the “brain unhealthy” components of the MIND diet and cognitive function adjusted for education level and total energy intake. Log transformed servings of red and processed meats were associated positively with episodic memory-delayed recall (β = 1.833, *p* = 0.008) and attention/working memory (β = 1.876, *p* = 0.035). While log transformed servings of fast and fried foods were negatively associated with phonemic fluency (β = −9.408, *p* = 0.024). Following adjustment for multiple comparisons, no associations remained significant with components of the MIND diet and cognitive function. Complete results from both unadjusted and adjusted linear regression analyses are presented in Supplemental Tables 6 and 7.

## Discussion

Disruptive cognitive side effects from cancer treatments impair quality of life for millions of cancer survivors. Our findings from a sample of postmenopausal breast cancer survivors, 3–12 months after completion of chemotherapy, suggest that each one-point increase in log transformed MIND diet score is associated with a 1.163-point higher global cognitive function. These findings are consistent with previous observational studies that have linked the MIND diet to reduced cognitive decline and a lower risk of neurodegenerative diseases [12–14]. Additionally, our results align with those of a randomized controlled trial among women with obesity, which found improvements in the domains of working memory, verbal recognition memory, and attention, among those that received a MIND diet intervention compared to the control group [14].

It was also observed that non-fried fish intake was associated with better global cognitive scores, attention/working memory, phonemic and semantic fluency. In non-cancer survivors, a systematic review of prospective research observed a 12% reduction in the relative risk of dementia of Alzheimer type with every 100 g/week increase in fish intake [33]. In our sample of breast cancer survivors, weekly servings of non-fried fish were also correlated with levels of DHA in the blood, which corroborates self-reported diet intake. Additionally, we found that blood levels of DHA were significantly correlated with several domains of cognitive function in the direction that would indicate better cognitive function. Blood levels of DHA has been shown to act as biomarker of fish and DHA intake in the diet [18] and is important in the resolution of inflammation [34]. In preclinical mouse models of chemotherapy, enrichment of the diet with EPA and DHA is associated with reduced neuroinflammation in the brain [35–37]. Furthermore, in a study of 1047 breast cancer survivors, it was observed that regular use of fish oil supplements, high in DHA, was associated with better delayed recall [38].

Among the “brain-healthy” components of the MIND diet, we observed beneficial associations between bean and legumes and other vegetable intake with cognitive performance. Both were linked to better phonemic fluency as measured by the COWAT. This is similar to findings from the Canadian Longitudinal Study on Aging, which associated low fruit and vegetable intake with poorer phonemic fluency performance [39]. Additionally, fiber intake, which is high in beans, legumes and vegetables, has been associated with reduced inflammation [40, 41]. Weekly servings of other vegetables were also correlated with higher levels of plasma β-carotene, although in this study, blood levels of β-carotene were not correlated with cognitive outcomes. In a cross-sectional analysis of the MIND trial, it was observed that α-carotene was higher in participants consuming more foods of the other vegetable category, which includes carrots, a source of α-carotene. Additionally, blood levels of this carotenoid were positively associated with higher levels of global cognition and semantic memory [42].

In terms of “brain-unhealthy” components two significant relationships were observed one negative relationship between fast and fried food and phonemic fluency, and positive relationships between red and processed meats intake with episodic memory-delayed recall and attention/working memory. The majority of the “brain unhealthy” foods are high in saturated fatty acids (SFA), which is limited in the MIND diet pattern due to adverse effects on cognitive function [12, 43]. Unexpectedly, the findings from our study indicated a positive association between weekly servings of red and processed meats and better performance on the test of delayed verbal recall and attention/working memory. This may be due to 90% of the sample consuming less than 4 servings of red and processed meat weekly, with the remaining 10% consuming less than 7 servings. This low intake of red and processed meat is within the ideal (< 4 servings/week) or moderate (4 to < 7 servings/week) range for the MIND diet pattern recommendations. This suggests that red and processed meat consumed within MIND diet recommendations may contribute to the neuroprotective potential of this diet.

This study had several strengths and limitations. To our knowledge, this is the first research to describe the association of the MIND dietary pattern to cognitive function in cancer survivors. Self-reported dietary data were collected via an electronic format that provides images and portion size to reduce potential measurement bias. Furthermore, nutritional biomarkers were measured and significant correlations with key self-reported food group components were observed. However, this study had several limitations, including the cross-sectional design, which prevents establishment of causality. In addition, the small sample size which identified primarily as white (90.48%), reduces generalizability to the diverse population of breast cancer survivors. Small sample size also limited the ability to include several demographic and clinical characteristics that could impact the association between diet and cognition among cancer survivors. Although nutritional biomarkers addressed some biases in self-reported dietary intake, there are other uncontrolled sources of error such as social desirability bias [44]. Cognitive tests were delivered remotely, which is not a fully validated method, however, testing was conducted following the IOPCR guidelines [23]. Additionally, certain blood biomarkers, like blood carotenoid levels, may be more reflective of compound bioavailability, and less reflective of absolute intakes from fruit and vegetables [19]. For these reasons, large randomized controlled trials are needed to better understand the potential of the MIND diet and components to prevent and manage negative cognitive side effects associated with cancer treatment.

## Conclusion

Among postmenopausal breast cancer survivors within one year post completion of chemotherapy, a better MIND diet pattern score was associated with better cognitive function as measured by an objective battery of cognitive tests. These findings suggest that adherence to the MIND diet pattern may provide protection of cognitive function in cancer survivors that have received chemotherapy. Future randomized controlled trials are needed to determine the potential of the MIND diet pattern to attenuate negative cognitive side effects that result from cancer and subsequent treatments.

## Supplementary Information

Below is the link to the electronic supplementary material.

Supplementary file1 (DOCX 76 KB)

## Data Availability

The datasets generated during and/or analyzed during the current study are available from the corresponding author on reasonable request.
