# Supplementary material for: The association of the MIND diet and its components with cognitive function in postmenopausal breast cancer survivors
Source: Support Care Cancer. 2025 Jul 31;33(8):736. doi: 10.1007/s00520-025-09789-9 (PMC12313734; doi:10.1007/s00520-025-09789-9)
Supplement: Supplementary file 1 — Supplementary file1 (DOCX 76 KB) [file 520_2025_9789_MOESM1_ESM.docx]

**Not Enrolled (n=89)**

Not Interested (n=36)

Ineligible (n=26)

Study Full (n=2)

No Response (n= 25)

**Completed Screener (n=118)**

**Consented & Enrolled (n=30)**

**Not included in Analysis (n=9)**

Incomplete cognitive testing (n=6)

Ineligible (n=1)

Energy Intake Implausible <600kcal (n=2)

**Complete Data (n=21)**

**Supplemental Figure 1:** Study Recruitment and Enrollment

**Supplemental Table 1: MIND Diet Components and Scoring ^a^**

| **Food Component** | **Unit** | **Score** | | |
| --- | --- | --- | --- | --- |
|  |  | **0** | **0.5** | **1** |
| **Green Leafy Vegetables** | servings/week | ≤ 2 | > 2 to < 7 | ≥ 7 |
| **Other Vegetables** | servings/week | < 5 | ≥ 5 to < 7 | ≥ 7 |
| **Berries** | servings/week | < 1 | ≥ 1 to < 5 | ≥ 5 |
| **Nuts** | servings/week | < 1 | ≥ 1 to < 5 | ≥ 5 |
| **Olive Oil** | Tbsp/day | < 1 | ≥ 1 to < 2 | ≥ 2 |
| **Whole Grains** | servings/day | < 1 | ≥ 1 to < 3 | ≥ 3 |
| **Non-fried Fish** | servings/week | < 1 | 1 | > 1 |
| **Beans and Legumes** | servings/week | < 1 | ≥ 1 to < 3 | ≥ 3 |
| **Poultry (not fried, skinless)** | meal/week | < 1 | ≥ 1 to < 2 | ≥ 2 |
| **Butter and stick margarine** | Tsp (pat)/day | ≥ 2 | > 1 to < 2 | ≤ 1 |
| **Regular Cheese** | servings/week | ≥ 7 | > 2 to < 7 | ≤ 2 |
| **Red and Processed Meats** | servings/week | ≥ 7 | ≥ 4 to < 7 | <4 |
| **Fast and Fried Foods** | meals/week | ≥ 4 | ≥ 1 to < 4 | <1 |
| **Pastries and Sweets** | servings/week | ≥ 7 | ≥ 5 to < 7 | <5 |

^a^ Scoring adapted from supplementary table 2 in Liu et al 2022.^1^

**Supplemental Table 2: Demographic and Clinical Characteristics for Median Split of MIND Diet Scores ^a^**

| **Variable** | **Low MIND (4.0 – 6.5)** | **High MIND (7.0 – 11.0)** | **P-value ^b^** |
| --- | --- | --- | --- |
|  | **Mean (SD)** | **Mean (SD)** |  |
| Age (years) | 56.73 (6.53) | 59.40 (8.45) | 0.42 |
| **Race** | **N (%)** | **N (%)** | **P-value** |
| Black or African American | 1 (9.09%) | 1 (10.00%) | 1.00 |
| White | 10 (90.91%) | 9 (90.00%) |  |
| **Education** | **N (%)** | **N (%)** | **P-value** |
| Less than a bachelor’s degree | 6 (54.55%) | 5 (50.00%) | 0.83 |
| Bachelor’s degree or advanced degree | 5 (45.45%) | 5 (50.00%) |  |
| **Employment** | **N (%)** | **N (%)** | **P-value** |
| Work 40+ hours a week | 7 (63.64%) | 3 (30.00%) | 0.02 |
| Work fewer than 40 hours a week | 1 (9.09%) | 0 (0.00%) |  |
| Homemaker | 2 (18.18%) | 0 (0.00%) |  |
| Retired | 1 (9.09%) | 2 (20.00%) |  |
| Unemployed | 0 (0.00%) | 5 (50.00%) |  |
| **Physical Activity Level** | **N (%)** | **N (%)** | **P-value** |
| Sedentary | 5 (45.45%) | 1 (10.00%) | 0.15 |
| Low Active | 5 (45.45%) | 5 (50.00%) |  |
| Active | 1 (9.09%) | 4 (40.00%) |  |
| Very Active | 0 (0.00) | 0 (0.00) |  |
| Extremely Active | 0 (0.00) | 0 (0.00) |  |
| **Breast Cancer Stage** | **N (%)** | **N (%)** | **P-value** |
| I | 3 (27.27%) | 3 (30.00%) | 0.86 |
| II | 4 (36.36%) | 5 (50.00%) |  |
| III | 4 (36.36%) | 2 (20.00%) |  |
| **HER-2 Status** | **N (%)** | **N (%)** | **P-value** |
| Positive | 6 (54.55%) | 0 (0.00%) | 0.012 |
| Negative | 5 (45.45%) | 9 (90.00%) |  |
| Unknown | 0 (0.00%) | 1 (10.00%) |  |
| **Treatment ^c^** | **N (%)** | **N (%)** | **P-value** |
| Surgery - lumpectomy/partial mastectomy | 3 (27.27%) | 2 (20.00%) | 1.00 |
| Surgery - total (simple) mastectomy | 3 (27.27%) | 2 (20.00%) | 1.00 |
| Surgery - modified radical mastectomy | 5 (45.45%) | 6 (60.00%) | 0.67 |
| Radiation therapy | 6 (54.55%) | 8 (80.00%) | 0.36 |
| Chemotherapy | 11 (100.00%) | 10 (100.00%) | 1.00 |
| Anti-Hormone or anti-estrogen therapy (e.g. Tamoxifen, Megace, Arimidex, Femara or other aromatase inhibitor) | 4 (36.36%) | 5 (50.00%) | 0.67 |
| Targeted therapy (e.g. HER2 inhibitors [Herceptin], CDK4/6 inhibitors [Ibrance], etc) | 5 (45.45%) | 1 (10.00%) | 0.15 |
| Immunotherapy (e.g. Keytruda, Tecentriq) | 4 (36.36%) | 3 (30.00%) | 1.00 |

^a^ Abbreviations: GED: General Education Development; HER2: Human Epidermal growth factor receptor 2; CDK: Cyclin-dependent kinase

^b^ P-values were calculated from comparison of low and high MIND categories via two-sample t-test for continuous variables and Fisher's exact test for categorical variables.

^c^ Treatment categories are not mutually exclusive, therefore total percentages sum to greater than 100%.

**Supplemental Table 3: MIND Diet Components Association with Nutritional Biomarkers ^a^**

| **Food Component** | **Alpha-tocopherol n=20** | | **Lycopene n=18** | | **Beta-Cryptoxanthin n=14** | | **Lutein n=20** | | **Zeaxanthin n=9** | | **Beta-carotene n=18** | | **Retinol n=20** | |
| --- | --- | --- | --- | --- | --- | --- | --- | --- | --- | --- | --- | --- | --- | --- |
|  | **rho** | **p** | **rho** | **p** | **rho** | **p** | **rho** | **p** | **rho** | **p** | **rho** | **p** | **rho** | **p** |
| **Green**  **Leafy**  **Vegetable** | 0.024 | 0.919 | -0.029 | 0.908 | -0.161 | 0.579 | 0.357 | 0.122 | 0.259 | 0.494 | 0.323 | 0.189 | -0.104 | 0.661 |
| **Other**  **Vegetable** | -0.062 | 0.795 | 0.261 | 0.292 | 0.020 | 0.946 | 0.331 | 0.153 | -0.233 | 0.539 | **0.480** | **0.045** | -0.361 | 0.118 |
| **Nuts** | 0.170 | 0.470 |  |  |  |  |  |  |  |  |  |  |  |  |
| **Whole**  **Grains** | 0.379 | 0.099 |  |  |  |  |  |  |  |  |  |  |  |  |
|  |  |  |  |  |  |  |  |  |  |  |  |  |  |  |
| **Food Component** | **EPA  n=21** | | **DHA n=21** | |  |  |  |  |  |  |  |  |  |  |
|  | **rho** | **p** | **rho** | **p** |  |  |  |  |  |  |  |  |  |  |
| **Non-fried fish** | 0.420 | 0.059 | **0.536** | **0.013** |  |  |  |  |  |  |  |  |  |  |

^a^ Bolded values indicate significant results p < 0.05

**Supplemental Table 4: Spearman Correlations of Nutritional Biomarkers with Cognition**

| **Cognitive Test** | **Beta-carotene n=18** | | **EPA n=21** | | **DHA n=21** | |
| --- | --- | --- | --- | --- | --- | --- |
|  | **rho** | **p** | **rho** | **p** | **rho** | **p** |
| **HVLT Sum Trials 1-3** | -0.212 | 0.394 | 0.299 | 0.187 | **0.600** | **0.005** |
| **HVLT Trial 4** | -0.060 | 0.810 | 0.039 | 0.866 | **0.434** | **0.050** |
| **DSF** | -0.272 | 0.273 | 0.338 | 0.133 | 0.197 | 0.390 |
| **DSB** | -0.329 | 0.182 | 0.060 | 0.793 | 0.271 | 0.232 |
| **OTMT-A** | **-0.549** | **0.020** | -0.077 | 0.738 | -0.246 | 0.280 |
| **OTMT-B** | -0.259 | 0.296 | -0.232 | 0.308 | **-0.461** | **0.036** |
| **COWAT (FAS)** | 0.169 | 0.500 | 0.2739 | 0.228 | **0.456** | **0.039** |
| **COWAT (Animals)** | 0.402 | 0.098 | 0.346 | 0.125 | **0.570** | **0.008** |
| **Global Cognitive Score** | 0.234 | 0.346 | 0.368 | 0.101 | **0.724** | **<0.001** |

Bolded values indicate significant results p<0.05.

**Supplemental Table 5: Association of Log Transformed MIND Diet Score with Cognitive Function ^a^**

| **Cognitive Variable** | **Unadjusted** | | **Adjusted** | |
| --- | --- | --- | --- | --- |
|  | **Estimate** | **p-value** | **Estimate** | **p-value** |
| **HVLT Trials 1-3** | 3.069 | 0.405 | 3.063 | 0.285 |
| **HVLT Trial 4** | **3.727** | **0.020** | **3.726** | **0.019** |
| **DSF** | **4.059** | **0.039** | **4.046** | **0.024** |
| **DSB** | 1.130 | 0.595 | 1.123 | 0.598 |
| **OTMT-A** | 0.292 | 0.828 | 0.289 | 0.837 |
| **OTMT-B** | -10.311 | 0.421 | -10.343 | 0.387 |
| **COWAT (FAS)** | **42.322** | **0.003** | **42.305** | **0.002** |
| **COWAT (Animals)** | 2.465 | 0.707 | 2.480 | 0.696 |
| **Global Score** | **1.163** | **0.040** | **1.163** | **0.013** |

^a^ Abbreviations: HVLT = Hopkins Verbal Learning Test; DSF = Digit Span Forward; DSB = Digit Span Backward; OTMT-A = Oral Trail Making Test Trial A; OTMT-B = Oral Trail Making Test Trial B; FAS = Controlled Oral Word Association Test FAS Trial. Bolded values indicate significant results p<0.05

^b^ Adjusted for level of education and total calorie intake

**Supplemental Table 6: Unadjusted Association of Log Transformed MIND Diet Score and Components with Individual Cognitive Tests ^a^**

| **Variable** | **Statistic** | **Global Score** | **HVLT 123** | **HVLT Trial 4** | **DSF Total** | **DSB Total** | **OTMT-A** | **OTMT-B** | **COWAT (FAS)** | **COWAT**  **(Animals)** |
| --- | --- | --- | --- | --- | --- | --- | --- | --- | --- | --- |
| **Green Leafy**  **Vegetables** | **β** | 0.185 | -0.124 | 0.331 | 0.027 | -0.340 | 0.084 | -6.638 | 7.504 | 2.919 |
|  | **p-value** | 0.291 | 0.910 | 0.512 | 0.966 | 0.589 | 0.833 | 0.070 | 0.100 | 0.121 |
| **Other**  **Vegetables** | **β** | **0.486** | 1.268 | **1.283** | 0.688 | -0.412 | -0.100 | **-9.630** | **13.577** | **5.103** |
|  | **p-value** | **0.021** | 0.360 | **0.035** | 0.375 | 0.607 | 0.844 | **0.036** | **0.015** | **0.028** |
| **Berries** | **β** | 0.172 | 0.592 | 0.663 | -0.235 | -0.416 | -0.385 | -4.839 | 3.389 | 1.118 |
|  | **p-value** | 0.201 | 0.485 | 0.079 | 0.621 | 0.391 | 0.204 | 0.090 | 0.350 | 0.455 |
| **Nuts** | **β** | 0.077 | 0.673 | -0.066 | 0.315 | 0.072 | -0.023 | -2.404 | 0.476 | 0.005 |
|  | **p-value** | 0.536 | 0.383 | 0.853 | 0.468 | 0.871 | 0.935 | 0.370 | 0.887 | 0.997 |
| **Olive Oil** | **β** | 0.205 | 0.862 | 0.601 | 0.759 | 0.630 | 0.055 | -0.302 | 5.088 | 0.606 |
|  | **p-value** | 0.097 | 0.271 | 0.089 | 0.075 | 0.156 | 0.848 | 0.913 | 0.125 | 0.665 |
| **Whole Grains** | **β** | 0.037 | -0.288 | -0.036 | -0.737 | -0.569 | -0.515 | -5.254 | 1.346 | 0.553 |
|  | **p-value** | 0.815 | 0.769 | 0.937 | 0.168 | 0.305 | 0.136 | 0.110 | 0.749 | 0.749 |
| **Non-fried Fish** | **β** | **0.323** | **1.470** | 0.475 | 0.547 | 0.059 | -0.306 | -4.330 | **6.577** | **3.462** |
|  | **p-value** | **0.005** | **0.048** | 0.179 | 0.202 | 0.895 | 0.274 | 0.099 | **0.039** | **0.006** |
| **Beans and Legumes** | **β** | 0.201 | 0.386 | 0.325 | 0.215 | -0.474 | -0.271 | -3.702 | **8.612** | 2.568 |
|  | **p-value** | 0.179 | 0.684 | 0.455 | 0.685 | 0.381 | 0.429 | 0.254 | **0.024** | 0.114 |
| **Poultry (not fried, skinless)** | **β** | 0.193 | 0.610 | **0.746** | 0.597 | 0.708 | -0.291 | 0.804 | 5.939 | -0.409 |
|  | **p-value** | 0.144 | 0.466 | **0.043** | 0.195 | 0.131 | 0.335 | 0.783 | 0.088 | 0.783 |
| **Butter and stick margarine** | **β** | 0.052 | -0.057 | -0.378 | -0.636 | 0.001 | -0.253 | -5.367 | 0.107 | 2.025 |
|  | **p-value** | 0.728 | 0.952 | 0.377 | 0.215 | 0.998 | 0.452 | 0.086 | 0.979 | 0.211 |
| **Regular Cheese** | **β** | **0.486** | **2.899** | 0.682 | 0.894 | 0.364 | -0.237 | -6.797 | 10.346 | 3.783 |
|  | **p-value** | **0.027** | **0.034** | 0.302 | 0.263 | 0.661 | 0.651 | 0.167 | 0.084 | 0.128 |
| **Red Meat and Processed Meat** | **β** | 0.411 | 2.134 | **1.698** | -0.216 | 1.524 | -0.382 | -0.516 | 4.987 | 3.136 |
|  | **p-value** | 0.078 | 0.146 | **0.007** | 0.798 | 0.066 | 0.483 | 0.922 | 0.439 | 0.230 |
| **Fast and Fried**  **Foods** | **β** | 0.019 | 0.814 | -0.185 | -0.459 | -0.622 | -0.310 | -2.577 | -4.690 | 1.853 |
|  | **p-value** | 0.893 | 0.337 | 0.639 | 0.332 | 0.196 | 0.312 | 0.382 | 0.193 | 0.212 |
| **Pastries and**  **Sweets** | **β** | 0.097 | 1.148 | -0.040 | -0.337 | -0.232 | -0.234 | -3.748 | -2.780 | 2.332 |
|  | **p-value** | 0.518 | 0.212 | 0.926 | 0.518 | 0.666 | 0.489 | 0.242 | 0.488 | 0.148 |

^a^ Abbreviations: HVLT = Hopkins Verbal Learning Test; DSF = Digit Span Forward; DSB = Digit Span Backward; OTMT-A = Oral Trail Making Test Trial A; OTMT-B = Oral Trail Making Test Trial B; FAS = Controlled Oral Word Association Test FAS Trial. Bolded values indicate significant results p<0.05.

**Supplemental Table 7: Education and Calorie Adjusted Association of Log Transformed MIND Diet Score and Components with Individual Cognitive Tests ^a^**

| **Variable** | **Statistic** | **Global Score** | **HVLT**  **123** | **HVLT**  **4** | **DSF Total** | **DSB**  **Total** | **OTMT**  **A** | **OTMT**  **B** | **COWAT (FAS)** | **COWAT**  **(Animals)** |
| --- | --- | --- | --- | --- | --- | --- | --- | --- | --- | --- |
| **Green Leafy**  **Vegetables** | **β** | 0.068 | -1.263 | 0.175 | 0.163 | -0.496 | 0.315 | -4.498 | 7.574 | 1.955 |
|  | **p-value** | 0.692 | 0.190 | 0.764 | 0.804 | 0.491 | 0.505 | 0.264 | 0.146 | 0.359 |
| **Other**  **Vegetables** | **β** | **0.410** | 0.396 | 1.285 | 0.944 | -0.561 | 0.084 | -7.373 | **14.069** | 4.274 |
|  | **p-value** | **0.039** | 0.743 | 0.057 | 0.229 | 0.524 | 0.886 | 0.128 | **0.021** | 0.091 |
| **Berries** | **β** | 0.149 | 0.279 | 0.822 | -0.005 | -0.504 | -0.362 | -3.741 | 3.849 | 0.120 |
|  | **p-value** | 0.286 | 0.730 | 0.071 | 0.993 | 0.391 | 0.348 | 0.256 | 0.377 | 0.946 |
| **Nuts** | **β** | 0.135 | 1.177 | -0.008 | 0.762 | 0.312 | 0.052 | -2.584 | 1.507 | -0.169 |
|  | **p-value** | 0.240 | 0.064 | 0.983 | 0.072 | 0.523 | 0.872 | 0.345 | 0.678 | 0.908 |
| **Olive Oil** | **β** | 0.123 | 0.241 | 0.508 | 0.749 | 0.582 | 0.132 | 1.698 | 4.117 | -0.210 |
|  | **p-value** | 0.280 | 0.714 | 0.180 | 0.073 | 0.218 | 0.676 | 0.532 | 0.241 | 0.884 |
| **Whole Grains** | **β** | -0.049 | -1.081 | -0.221 | -0.615 | -0.728 | -0.589 | -4.601 | 1.435 | -1.171 |
|  | **p-value** | 0.783 | 0.281 | 0.713 | 0.358 | 0.324 | 0.221 | 0.268 | 0.796 | 0.598 |
| **Non-fried Fish** | **β** | **0.346** | 1.129 | 0.501 | **1.150** | 0.023 | -0.283 | -2.062 | **8.778** | **3.731** |
|  | **p-value** | **0.009** | 0.162 | 0.299 | **0.025** | 0.970 | 0.477 | 0.548 | **0.039** | **0.027** |
| **Beans and Legumes** | **β** | 0.175 | 0.025 | 0.298 | 0.557 | -0.519 | -0.182 | -2.039 | **10.168** | 2.068 |
|  | **p-value** | 0.226 | 0.976 | 0.548 | 0.314 | 0.397 | 0.653 | 0.558 | **0.016** | 0.253 |
| **Poultry (not fried, skinless)** | **β** | 0.143 | 0.248 | 0.680 | 0.529 | 0.647 | -0.273 | 1.762 | 5.252 | -0.824 |
|  | **p-value** | 0.211 | 0.710 | 0.070 | 0.223 | 0.174 | 0.392 | 0.522 | 0.135 | 0.571 |
| **Butter and stick margarine** | **β** | -0.081 | -1.082 | -0.864 | -0.655 | 0.092 | -0.137 | -3.905 | -1.776 | 1.115 |
|  | **p-value** | 0.620 | 0.239 | 0.105 | 0.285 | 0.894 | 0.762 | 0.308 | 0.728 | 0.585 |
| **Regular Cheese** | **β** | 0.377 | 2.024 | 0.481 | 1.601 | 0.360 | 0.046 | -1.427 | 11.489 | 1.959 |
|  | **p-value** | 0.135 | 0.161 | 0.582 | 0.091 | 0.741 | 0.948 | 0.817 | 0.143 | 0.545 |
| **Red Meat and Processed Meat** | **β** | 0.351 | 1.700 | **1.833** | 0.003 | **1.876** | -0.242 | 3.377 | 4.258 | 1.982 |
|  | **p-value** | 0.108 | 0.179 | **0.008** | 0.997 | **0.035** | 0.698 | 0.528 | 0.544 | 0.482 |
| **Fast and Fried**  **Foods** | **β** | -0.152 | 0.098 | -0.547 | -0.540 | -0.977 | -0.250 | 0.538 | **-9.408** | 0.822 |
|  | **p-value** | 0.286 | 0.905 | 0.252 | 0.317 | 0.092 | 0.526 | 0.875 | **0.024** | 0.647 |
| **Pastries and**  **Sweets** | **β** | -0.066 | 1.061 | -0.555 | -0.081 | -0.394 | -0.057 | -0.179 | -10.581 | 1.697 |
|  | **p-value** | 0.745 | 0.357 | 0.415 | 0.917 | 0.644 | 0.920 | 0.970 | 0.081 | 0.502 |

^a^ Abbreviations: HVLT = Hopkins Verbal Learning Test; DSF = Digit Span Forward; DSB = Digit Span Backward; OTMT-A = Oral Trail Making Test Trial A; OTMT-B = Oral Trail Making Test Trial B; FAS = Controlled Oral Word Association Test FAS Trial. Bolded values indicate significant results p<0.05.

**References**

1. Liu X, Morris MC, Dhana K, et al. Mediterranean-DASH Intervention for Neurodegenerative Delay (MIND) study: Rationale, design and baseline characteristics of a randomized control trial of the MIND diet on cognitive decline. Contemp Clin Trials. Mar 2021;102:106270. doi:10.1016/j.cct.2021.106270
